# The Quest for Arterial Recanalization in Acute Ischemic Stroke-The Past, Present and the Future

**DOI:** 10.4021/jocmr1342w

**Published:** 2013-06-21

**Authors:** Leonard L.L.Yeo, Vijay K Sharma

**Affiliations:** a Division of Neurology, National University Hospital, Singapore and Yong Loo Lin School of Medicine, National University of Singapore, Singapore

**Keywords:** Stroke, Thrombolysis, Intervention, Devices, Recanalization, Reperfusion

## Abstract

Ischemic stroke is one of the major causes of mortality and long-term disability. In the recent past, only very few treatment options were available and a considerable proportion of stroke survivors remained permanently disabled. However, over the last 2 decades rapid advances in acute stroke care have resulted in a corresponding improvement in mortality rates and functional outcomes. In this review, we describe the evolution of systemic thrombolytic agents and various interventional devices, their current status as well as some of the future prospects. We reviewed literature pertaining to acute ischemic stroke reperfusion treatment. We explored the current accepted treatment strategies to attain cerebral reperfusion via intravenous modalities and compare and contrast them within the boundaries of their clinical trials. Subsequently we reviewed the trials for interventional devices for acute ischemic stroke, categorizing them into thrombectomy devices, aspiration devices, clot disruption devices and thrombus entrapment devices. Finally we surveyed several of the alternative reperfusion strategies available. We also shed some light on the controversies surrounding the current strategies of treatment of acute ischemic stroke. Acute invasive interventional strategies continue to improve along with the noninvasive modalities. Both approaches appear promising. We conducted a comprehensive chronological review of the existing treatments as well as upcoming remedies for acute ischemic stroke.

## Introduction

Ischemic stroke (IS) is a major cause of mortality and long-term disability [1]. In past, only few treatment options were available and considerable proportion of stroke survivors remained permanently disabled. However, the past 2 decades witnessed rapid advances in acute stroke care. Since acute occlusion of an intracranial artery is responsible for the clinical manifestations, achieving timely recanalization remains the main aim of acute stroke care [2]. Fast dissolution of thrombi and arterial recanalization in acute IS often leads to dramatic clinical recovery [3].

Thrombolytic therapy with intravenously-administered tissue plasminogen activator (IV-tPA) remains the mainstay in acute IS. However, many interventional strategies have been attempted, with variable success, for rapid intracranial arterial recanalization and improve outcomes. In this review, we describe the evolution of systemic thrombolytic agents and various interventional devices, their current status as well as some of the future prospects.

## Thrombolytic Agents

Thrombolytic drugs activate plasminogen to plasmin, which in turn degrades fibrin and its derivatives. Although many thrombolytic agents have been tried in acute IS, only a few studies have performed direct comparisons regarding their efficacy and safety. Some of these agents are:

### Streptokinase

Tillett and Garner (1933) discovered that certain strains of hemolytic bacteria could dissolve fibrin clots. Later, Tillett purified streptokinase for intravenous use [4]. Streptokinase, a protein derivative from group C beta-hemolytic streptococci, works by activation of plasmin through fibrin-dependent as well as fibrin-independent mechanisms. However, being a bacterial protein, streptokinase often resulted in fever and hypotension. Although, these problems could be overcome with low-dose streptokinase, frequent intracranial and systemic hemorrhages due to an intense systemic proteolytic state limited its widespread use [5]. Nonetheless it is important to our current understanding of thrombolysis.

### Urokinase

Macfarlane and Pilling described the fibrinolytic potential of human urine in 1947. However, Sobel et al isolated the active constituent and named it urokinase (UK) [6]. UK is a serine protease with half-life of 14 minutes. Although, it activates plasminogen directly and does not evoke the antigenic response, UK lacks the fibrin selectivity and causes excessive risk of bleeding due to a severe lytic state. It has not been used much in clinical practice due to its poor availability.

### Tissue plasminogen activator (tPA)

Alteplase, this 527-amino acid single-chain serine protease is a naturally occurring fibrinolytic agent produced by endothelial cells. It is a powerful agent with fibrin specificity, binds to the fibrin surface causing a conformational change and accelerating the conversion of plasminogen to plasmin to dissolve the thrombus. Since it does not activate plasminogen that is freely floating in the blood, it does not lead to a severe lytic state. However, tPA activates N-methyl-D-aspartate (NMDA) receptors in the neuronal cell-death pathway, amplifies calcium conductance and activates extracellular matrix metalloproteinases [7]. These mechanisms often result in the breakdown of the blood brain barrier and development of intracranial bleeds as well as worsening of the cerebral oedema associated with acute IS. Commercially used tPA, produced using recombinant technology, is approved for acute myocardial infarction, acute stroke, pulmonary embolism and central venous catheter thrombotic occlusion.

### Pro-urokinase

A precursor of urokinase (pro-UK) has a half-life of 7 minutes with less side effects compared tPA. It was used intra-arterially in the PROACT trial.

### Tenecteplase

It is considered a third generation thrombolytic agents with favorable arterial recanalization rates. It is an improvement upon tPA at the molecular level by modification of three enzymatic sites to increase fibrin specificity, less depletion of fibrinogen and prolonging its half-life [8].

### Reteplase

The development of Reteplase began from the hypothesis that a decreased affinity to hepatocytes could prolong the half-life of the circulating drug and enable single bolus administration. Accordingly, several deletions of domains of the tPA molecule resulted in Reteplase molecule with reduced fibrin-binding activity and a fourfold increase in the plasma half-life (18 minutes vs 4 minutes for tPA). Although synthesized by E. coli, Reteplase is not immunogenic. Some of the salient differences between streptokinase, alteplase and reteplase are shown in Table 1.

**Table 1:** Comparison of the Properties of Streptokinase, Alteplase and Reteplase

	Streptokinase	Alteplase	Reteplase	Desmoteplase
Molecular weight	47k daltons	70k daltons	39k daltons	52k daltons
Half-life	23 mins	Less than 5 mins	13 - 15 mins	4 hours
Plasminogen activation	Indirect binding	Direct binding	Direct binding	Direct binding
Fibrin selective	No	Yes	Yes	Fibrin specific
Allergic response	Yes	No	No	Yes
Administration	Infusion	Infusion	bolus	Bolus

### Desmoteplase and ancrod

These compounds are genetically engineered agents, with fibrinolytic properties, as well as prothrombotic effects via the production of thrombin during thrombolysis, and subsequent activation of platelets and fibrinogen. Desmoteplase is a non-neurotoxic agent which is made from vampire bat venom and is a longer acting thrombolytic with greater fibrin specificity than tPA. Ancrod (Viprinex) is an enzyme derived from pit viper venom with defibrinating properties.

## Intravenous Thrombolysis With tPA

Intravenous tPA is the current standard of care and new modalities are benchmarked against it. Intravenous fibrinolytic therapy with tPA within the first 3 hours of IS onset offers substantial benefits with potentially disabling deficits, while in the 3 to 4.5 hour window, the benefits are more modest[9, 10]. The National Institute of Neurological Disorders and Stroke (NINDS) trials included patients treated within 3 hours of their symptom onset [9]. Alteplase ThromboLysis for Acute Noninterventional Therapy in Ischemic Stroke (ATLANTIS) trial included patients up to 6 hours of stroke-onset in part A and in the 3 - 5 hour time window in part B [11]. European Cooperative Acute Stroke Study 1 and 2 recruited patients up to 6 hours [12, 13]. The pooled analysis of these 6 clinical trials revealed improved outcomes, if treatment was initiated within 90 minutes (Odds ratio (OR) 2.8, 95% Confidence interval (CI) 1.8 - 4.5). However, the potential benefit existed even beyond 3 hours (OR 1.4, 95% CI 1.05 - 1.85 for 180 - 270 minutes) [14]. This led to the initiation of the European Cooperative Acute Stroke Study (ECASS III) which was a double-blind randomized placebo-controlled trial [15]. It was designed to evaluate acute IS treated with IV-tPA within 3 - 4.5 hours and recruited 821 patients from 19 European countries. Treated at a median time of 3 hours 59 minutes, patients receiving IV-tPA had favourable outcomes compared to placebo (52.4% versus 45.2%; OR 1.34; 95% CI 1.02 - 1.76; P = 0 .04). Although the incidence of symptomatic intracranial hemorrhage (SICH) was higher with tPA (2.4% versus 0.2%; P = 0 .008), mortality did not differ significantly between the two groups (7.7% versus 8.4%; P = 0 .68) [15]. The controversy about the efficacy of tPA in acute IS patients older than 80 years of age was evaluated by the International Stroke Trial III (IST-III) [16] in 3035 patients and concluded that in patients older than 80 years of age, IV-tPA was as efficacious as in patients less than 80 years of age.

### IV-tPA and recanalization rates

Recanalization rates vary depending on the site of occlusion and clot burden. A meta-analysis of patients treated with IV thrombolysis showed higher recanalization rates (46%) at 24 hours compared to the spontaneous recanalization rate (24%) [2]. While IV-tPA recanalizes only 10% of distal carotid occlusions, recanalization may occur in up to 33% patients with proximal MCA occlusion. Low rates of recanalization have also been found in patients with coexisting carotid artery disease and diabetes mellitus [17].

Although, recanaliztion of an occluded artery remains the primary aim of systemic thrombolysis, clinical outcomes, especially early recovery, might depend on the timing of recanalization. In a recent study an Asian patient cohort of 240 acute IS patients, treated with IV-tPA, we demonstrated early recanalization (by transcranial Doppler monitoring) within first 2 hours of treatment initiation was associated with favorable functional outcome (mRS 0-1) at 3 months (OR 3.048; 95%CI 1.537 - 6.046, P = 0.001) [18].

## Intravenous Thrombolysis With Desmoteplase

The desmoteplase in acute IS trial (DIAS) was a dose finding trial targeting patients with a perfusion-diffusion mismatch on magnetic resonance imaging (MRI) done within 3 to 9 hours of symptom-onset [19]. Initially patients were randomized to IV desmoteplase 25 mg, 37.5 mg, or 50 mg versus placebo, however the rate of symptomatic intracranial hemorrhage was high in the desmoteplase arm and the study team decided to reduce the doses and based it on body weight (62.5 µg/kg, 90 µg/kg, and 125 µg/kg). The study found that clinical improvement was associated with early reperfusion. Overall 54.3% patients treated in 3 - 6 hours and 40% in 6 - 9 hour time window showed reperfusion, that was significantly correlated with favorable clinical outcome (P = 0.0028).

In the dose escalation of desmoteplase in acute stroke (DEDAS) trial, the randomized dose-escalation study in patients who presented within 3 to 9 hours of stroke onset with MRI perfusion-diffusion mismatch [20]. The results of DEDAS were consistent with the results of DIAS. At a dose of 125 µg/kg desmoteplase appeared to improve clinical outcome, especially in patients fulfilling all MRI criteria. Subsequently, a phase III study (DIAS-2) was conducted to confirm the results of the DIAS/DEDAS studies and also to investigate the clinical efficacy and safety of desmoteplase in patients with acute IS with tissue at risk (at least 20% ischemic-pneumbra brain tissue) by using diffusion weighted imaging/perfusion weighted imaging (DWI/PWI) mismatch techniques 3 - 9 hours after stroke onset [21]. This study comprised 186 patients that were randomized into a placebo-controlled dose-ranging trial investigating the efficacy and safety of 2 doses of desmoteplase (90 µg/kg and 125 µg/kg), given as an IV bolus. The median baseline National Institute of Health Stroke Scale (NIHSS) score was 9 points and only 30% of the recruited patients demonstrated a visible arterial occlusion at presentation. Hence, the core lesion and the mismatch volumes were small (median volumes were 10.6 cm^3^ and 52.5 cm^3^, respectively). There was no significant difference in the 3 groups according to the improvement on the NIHSS score. Mortality was 6.3% in the placebo group, 5.3% in the 90 µg/kg group, and 21.2% in the 125 µg/kg group. SICH was noted in 0%, 3.5%, and 4.5% of patients, respectively. It is believed that the DIAS-2 study failed to show a significant benefit of desmoteplase due to the low baseline NIHSS score and small mismatch volumes. However, when the DIAS-2 data were analyzed with a more conservative definition of mismatch (PWI-DWI volume > 75 mL), beneficial effects of desmoteplase were apparent [21].

## Intravenous Thrombolysis With Ancrod

The Stroke Treatment with Ancrod Trial (STAT) evaluated patients who presented with 3 hours of onset of stroke [22]. It was a randomized, multicentre, parallel, double-blind, placebo-controlled trial held in the United States and Canada. A total of 500 patients were randomly assigned to receive Ancrod (n = 248) or placebo (n = 252) as a continuous 72-hour IV-infusion, beginning within 3 hours of stroke onset, followed by infusions lasting 96 and 120 hours. The trial showed favorable outcome in patients treated with Ancrod versus placebo (42.2% versus 34.4%). Although there was a trend toward more SICH in the Ancrod group (5.2% versus 2.0%), it did not reach statistical significance. Phase-III trials with Ancrod (ASP I and II) attempted to use Ancrod within 6 hours of stroke onset but had to be stopped prematurely due to increased SICH. The post-trial analysis suggested that modifying the dose of Ancrod could reduce SICH and improve the outcomes [23].

## Glycoprotein (GP) IIIb/IIa Antagonists

Abciximab and Tirofiban are the two GPIIb/IIIa receptor antagonists that have been tried in acute IS. Abciximab shares binding affinity for the vascular integrin a_v_ b_3_ and the leukocyte integrin a_M_ b_2_. Abciximab exerts high binding affinity for GPIIb/IIIa. A small bolus blocks approximately 80% of the receptors and induces > 80% blockade of ADP-induced platelet aggregation. It dissociates slowly from the receptors over 30 min. In contrast, tirofiban has a lower binding affinity for the receptor and dissociates more rapidly. Hence, it depends on higher plasma levels to sustain a target receptor blockade level above 80%.

Abciximab is a chimeric mouse/human monoclonal antibody which binds to the platelet glycoprotein IIb/IIIa Receptor. It has been mostly used as an adjunct to endovascular procedures. In a study on 400 patients with acute IS treated with Abciximab within 6 hours of onset (AbESTT), it seemed to have a lower risk of SICH. This favourable profile was also seen in a dose escalation study comprising 74 subjects that were treated up to 24 hours after symptom-onset [24]. Despite these promising preliminary studies, the Abciximab in Emergency Treatment of Stroke Trial Abciximab (AbESTT-II), a phase III trial, where 221 patients were randomized to abciximab and 218 to placebo, showed no significant difference in efficacy and functional independence at 90 days between the drug and placebo groups. The trial was terminated prematurely due to an unfavourable benefit-risk profile as the incidence of fatal or SICH was significantly higher in the Abciximab-treated group at discharge/Day 5 and 90 days [25].

The Reopro Retevase Reperfusion of Stroke Safety Study (ROSIE) was an open-label, dose escalation, safety and proof-of-principle study looking into the safety and efficacy of Abciximab (ReoPro) and Reteplase (Retevase) in reperfusion the ischemic tissue [26]. It included acute IS patients within 3-24 hours of symptom-onset, with a disabling stroke, NIHSS score ≤ 16 points, and evidence of a perfusion deficit on PWI/MRI and MR angiography. Although Abciximab was safe, it was not effective in re-establishing blood flow. However, its combination with Reteplase showed acceptable safety and efficacy.

The Safety of Tirofiban in Acute Ischemic Stroke (SaTIS) was a phase 2 study designed to evaluate safety and clinical efficacy [27]. Patients were randomized to either 48-hours of placebo (n = 123) or Tirofiban infusion (n = 127). The trial reported an increased risk of bleeding associated with Tirofiban that did not reach statistical significance. At 5 month follow up, there was no significant difference in functional improvement but significant mortality benefit was observed for the Tirofiban group over placebo.

## Ultrasound Enhancement of Thrombolysis With IV-tPA (Sonothrombolysis)

Ultrasound waves in the low MHz-kHz frequency have the ability to enhance the thrombolytic effect of IV-tPA [28]. Various conformational changes such as reversible disaggregation of uncross linked fibrin fibers and microcavity formation in the shallow layers of thrombus lead to increased penetration of tPA into the clot, resulting in residual flow enhancement with microstreaming and vessel dilation.

CLOTBUST was a phase II trial that recruited126 patients with acute IS treated with IV-tPA, randomly assigned to receive 2 Mhz continuous transcranial Doppler (TCD) monitoring or placebo [29]. Complete recanalization or dramatic clinical recovery within 2 hours after the administration of tPA bolus occurred in 49% in the target group as compared to 30% in the control group (P = 0.03). There was no increase in the incidence of SICH.

In the TRUMBI trial, Daffertshofer et al [30], included 26 patients within the 6 hours time window in a multicenter clinical trial that evaluated 90 min of low frequency (300 kHz) ultrasound exposure. The study was stopped prematurely due to an increased incidence of SICH (tPA group 42% versus tPA plus ultrasound group 93%). Although no definite mechanisms were elucidated, it was believed to be due to reverberations of the long wavelength ultrasound, inside the head, leading to resonance creating several hotspots in addition to the mechanical damage and distortion of brain microvessels with kHz frequencies [30].

Synthetic microbubbles were initially used as contrast agents to improve the quality of ultrasound images. These microbubbles were believed to have a potential of enhancing the efficacy of thrombolytic agents [31]. Molina et al first tested galactose-based air microbubbles (Levovist^®^, Schering, Berlin, Germany) in humans with acute IS treated with IV-tPA [32]. Newer generation bubbles use phospholipid molecules that, when exposed to mechanical agitation, arrange themselves in nano-bubbles of consistent 1.2 µm (or even lesser) diameter [33]. As the injected bubbles approach and permeate through the thrombus, they are activated by ultrasound pressure wave, which breaks up the phospholipid shell and releases the gas. The thrombus surface undergoes cavitation from the gas that increases the surface area for thrombolytic action by tPA and accelerates lysis of clots. In a recent trial, higher re-canalization rates were seen with third-generation perflutren-lipid microspheres. The perflutren microspheres (µS) reached and permeated beyond intracranial occlusions, with no increase in symptomatic hemorrhage after systemic thrombolysis [34].

## Intra-Arterial (IA) Thrombolysis

Compared with IV administration, the IA approach to thrombolysis has some obvious advantages. It allows for higher concentrations of the thrombolytic agents to be administered locally close to or into the clot without inducing excessive systemic thrombolysis and reducing the risk of SICH. IA therapy has the advantage of better dose adjustment with angiographic direct visualization of clot lysis and avoids any thrombolytic exposure if no thrombus is seen. Since vascular access has already been achieved, it provides the possibility of mechanical disruption to facilitate thrombolysis or an avenue to switch to mechanical devices for clot retrieval and lysis if necessary as compared to IV thrombolysis where no vascular access has been attained. The smaller quantity of thrombolytic agent used with IA therapy makes treatment possible in patients with contraindications to systemic thrombolysis and minimizes the risk of systemic bleeding. The major disadvantages of IA therapy for acute IS include the delay in initiating thrombolysis. Like all other cerebrovascular interventions, IA thrombolysis is limited by the severe shortage of neurointerventionalists.

There have been no large head-to-head randomized trials that compared IV-tPA versus IA thrombolysis in acute IS. In open clinical series, IA thrombolysis has shown higher early re-canalization rates (50% to 80%) than IV therapy (30% to 50%) [35]. The PROACT (Prolyse in Acute Cerebral Thromboembolism) I and II trials randomized patients to IA thrombolysis or to conservative therapy. The PROACT I trial randomized 40 patients with MCA occlusions to either IA prourokinase or placebo within 6 hours of symptom-onset. Cerebral angiography was performed and M1 or M2 occlusions were treated with 6 mg of rpro-UK (n = 26) or placebo (n = 14). All patients received a concomitant heparin bolus followed by a 4-hours infusion. The final end points were recanalization efficacy at the end of the infusion period and neurologic deterioration from ICH within 24 hours of treatment. Partial or complete recanalization was seen in 57.7% of patients with IA thrombolysis compared with 14.3% treated with placebo. SICH occurred in 15.4% (4 of 26) thrombolysis patients and in 7.1% of the placebo patients. The number of patients included in this trial was too small to show statistical significance in clinical outcomes. However, the 90-day mortality was 29.6% with IA thrombolysis compared with 42.9% with placebo. Overall, PROACT I was the first organized trial proving the safety and efficacy of IA thrombolysis for the management of acute IS.

The only IA randomized trial, Prolyse in acute cerebral thromboembolism II (PROACT II), showed that treatment initiated up to 6 hours after onset of an MCA M1 or M2 occlusion was beneficial in acute IS [36]. The study randomized 121 patients to IA pro-urokinase and low-dose IV heparin, and the control group onsisted of 59 patients treated with low-dose IV heparin alone. The recanalization rates were significantly higher (66% vs 18%, P < 0.001) with a rate of favorable outcome at 90 days which was significantly higher in prourokinase than in the placebo group (40% vs. 25%, P = 0.04). The improved clinical outcomes were better in spite of the higher rate of SICH (10% vs. 2%). IA-tPA may therefore be an option in select patients with large artery occlusions, which have a poorer response to IV-tPA. Although the recanalization rates were better, the clinical benefit may be reduced by the time delay in initiation of the procedure and by the time necessary for chemical clot lysis. PROACT II trial was encouraging and demonstrated that the use of IA thrombolysis in acute ischemia of the anterior circulation leads to radiographic and clinical improvement. The Middle Cerebral Artery Embolism Local Fibrinolytic Intervention Trial (MELT), Japanese group investigated the IA administration of Urokinase in the setting of MCA stroke within 6 hours of onset [37]. A total of 114 patients underwent randomization. Although the study showed favorable 90-day functional outcome in the Urokinase treated patients with respect to controls group (49.1% versus 38.6%), this did not reach a significant level (P = 0.345). There were significantly more patients with NIHSS scores of 0 or 1 at 90 days in the urokinase group (P = 0.017). This randomized trial might have provided additional evidence for the efficacy of IA thrombolysis. However, it was aborted prematurely following the approval of IV-tPA in Japan for the treatment of acute IS.

Although the American Heart Association/American Stroke Association guidelines for the early management of IS recommend off-label IA therapy for selected patients who present within 6 hours of onset and are not candidates for IV tPA, there are no approved drugs for IA stroke therapy. When planning IA, the operator should record the time required to perform the procedure. Considering that the average intervention time varies from 45 to 180 minutes, high-risk patients should be treated within 4 to 5 hours from ischemia onset. Theron and colleagues investigated the efficacy of IA thrombolysis in relation to the site of arterial occlusion [38]. They recommended that IA fibrinolysis of the MCA should be performed within 6 hours if the occlusion involves the horizontal segment of MCA and the lenticulostriate arteries since the risk of hemorrhagic complication increases significantly beyond this. However, if the occlusion does not involve the horizontal MCA segment and the lenticulostriate arteries, then the treatment window can be extended to 12 hours following symptoms.

Basilar artery (BA) occlusion is a life-threatening event that poses a significant therapeutic challenge. The natural course of untreated BA occlusion has mortality rates ranging from 86% to 100%. In a series of about 300 patients, Furlan and Higashida reported IA recanalization rates of 60%. Patients with at least partial recanalization had reduced mortality (about 31%) as compared to 90% in non-recanalized patients [39]. Lindsberg and Mattle compared IV versus IA thrombolysis in BA occlusion and reported higher recanalization rates with the latter (65% vs 53%). Compared to 2% among the untreated patients, good functional outcome was noted in 22% of the thrombolyzed patients. However, the rates of dependency or death were equal between the two groups (76% to 78%) [39]. Therefore, emergent thrombolysis via either route of administration is of paramount importance to the survival of this high-risk patient population. A meta-analysis of multiple case series comparing IV and IA fibrinolysis for acute vertebrobasilar stroke showed marginally better recanalization rates with intra-arterial therapy (65% versus 53%; P = 0.05).[40] Similar findings were demonstrated by the Basilar Artery International Cooperative Study (BASICS) registry [41].

## Mechanical Thrombolysis

Mechanical thrombectomy is a therapeutic option for acute IS patients that present late or ineligible or fail IV thrombolysis. Mechanical disruption of the arterial clot has several advantages to IA thrombolysis. Endovascular methods comprise a wide range of mechanical devices designed to mechanically retrieve, fragment or penetrate thrombosis, atherosclerotic plaque and other vascular occlusions and is emerging as a viable alternative to chemical thrombolysis [42, 43]. Mechanical therapies can achieve recanalization much faster than the 2 hours required with IA thrombolysis. They are more effective in removing large thrombi in proximal vessels, may be more efficacious at achieving full recanalization and are associated with lower hemorrhage risk. However, in addition to the highly trained personnel required for technical skills needed for the endovascular navigation, the mechanical methods are associated with some inherent risks. Bulky catheters often require larger access sheaths, leading to the risk of bleeding complications at the arterial puncture site. The endovascular trauma to the blood vessel could cause endothelial damage and vessel rupture, especially in elderly patients. Finally, the dislodged clot material could embolise distally, exposing the already compromised circulation to additional ischemic risks. In a systematic review of studies of clot removal devices comprising laser, rheolytic, aspiration, coil, snare and ultrasonic devices, arterial recanalization was achieved in 68% and hemorrhage occurred in 23% [44]. The review suggests that mechanical embolectomy improves survival and functional outcomes when compared with no intervention. However, 29% patients died after the endovascular procedure and only 34% achieved favorable outcome at 90 days. The techniques of mechanical recanalization, with or without concomitant lysis, are becoming more refined and effective in rapid restoration of blood flow to salvageable brain tissue to minimize brain damage and improve survival rates [45].

## Endovascular Thrombectomy

Various devices have been tried to extract the blood clots from the intracranial arteries and achieve recanalization. These devices apply a constant force to the clot at its proximal or distal end. A griping or grasping attachment is typically used at the proximal end of the clot while ‘basket’ or ‘carrier’ is deployed at the distal end of the clot. One of the major aims is to minimize the risk of embolization during the procedure. Some of the most widely used examples are the Merci retriever (Concentric Medical), Neuronet device (Guidant, Santa Clara, CA), Phenox clot retriever (Phenox, Bochum, Germany), Catch thrombectomy device (Balt Extrusion, Montmorency, France) and the Alligator retrieval device (Chestnut Medical Technologies, Menlo Park, CA) [46-49].

## MERCI Retrieval System

The Merci Retrieval System (Concentric Medical) consists of an intra-arterial delivered corkscrew-shaped flexible nitinol (nickel titanium) wire retriever that traverses and ensnares the thrombus. The Mechanical Embolus Removal in Cerebral Ischemia (MERCI) trial was a prospective, nonrandomized, single arm trial designed to evaluate the safety and technical efficacy of mechanical embolus removal within 8 hours of stroke onset. A total of 28 patients with severe stroke (median baseline NIHSS score 19 points) underwent Merci clot retrieval and the results were compared against the data from PROACT II trial’s control arm [46, 49]. Overall recanalization was achieved in 48% of patients as compared with the 18% spontaneous revascularization rate in the control arm of the PROACT II trial. Procedural complications occurred in 7.1% of patients while SICH were observed in 7.8% with an overall 44% mortality. Significantly more patients with successful recanalization achieved favorable outcome (modified Rankin score; mRS ≤ 2) at 90 days as compared to persistent occlusion (46% versus 10%; P < 0.0001). Similarly, mortality was lower in revascularized patients (31.8%) as compared to the patients with persistent arterial occlusion (54.2%). The Merci device was approved in 2004 for endovascular clot retrieval in acute IS [50].

The Multi-MERCI trial enrolled 177 patients from 15 centers with the inclusion criteria similar to the first MERCI trial. Additionally, patients treated with IV-tPA and failed recanalizations were also included. Overall recanalization with the device was achieved in 55% patients, with a procedure-related complication rate of 5.5%. Adjunctive IA thrombolytic administration, employed after device failure, improved the overall final recanalization rates to 68%. The trial reported good functional outcome (mRS ≤ 2) at 90 days in 36% and mortality in 34% of the study patients. Again, the mortality rates (25%) were significantly lower in the successfully recanalized group versus 52% in the failures. Symptomatic ICH occurred in 9.8% of patients, with 2.4% having large hemorrhages. Importantly, no significant increase was observed in SICH rates or procedural complications in patients treated with IV-tPA prior to the use of Merci device [51].

## Other Mechanical Devices

Neuronet device is a flexible and tapered core wire with a flexible retrieval basket attached to its distal tip. Its use was described in 2002 for vertebrobasilar occlusions. The micro-guide-wire-based device has a self-expanding basket that can be pushed through a standard microcatheter and used to retract an embolus [52].

The Catch Device was recently evaluated in a study of 40 patients. Recanalization was achieved in 65% patients. However, 18% patients developed SICH. The study reported favorable functional outcome (mRS ≤ 2) in 39% and mortality in 41% of the patient cohort [53]. The alligator retrevial device was also evaluated recently in a small cohort of 7 patients and was able to remove the clot in a majority of cases [54].

The Phenox Clot Retriever device consists of a highly flexible nitinol/platinum alloy core wire compound that uses perpendicularly oriented polyamide microfilaments arranged in a brush-like configuration and a proximal self-expanding nitinol basketlike cage that reduces thrombus compression with a consequently high recanalization and low complication rate. The device is attached to the corpus of a microguidewire and comes in different sizes for vessels ranging from 1 to approximately 4 mm. It is deployed distal to the clot and is slowly pulled back under continuous aspiration via the guiding catheter. Merci, Phenox and Catch devices have almost similar efficacy for clot mobilization and retrieval.

## Thrombus Disruption

Some devices have been used for mechanical disruption of the clot by a micro-guidewire or a snare and restore the blood flow. The examples include the Endovascular Photoacoustic Recanalization device or EPAR (Endovasix, Belmont, CA) and the LaTIS Laser device (LaTIS, Minneapolis, MN). In a cohort of 34 patients with a median NIHSS of 19 points, the EPAR device demonstrated a recanalization rate of 41.1%. SICH was observed in 2 (5.9%) patients and the mortality rate was 38.2% [55]. However, the potential endothelial damage with resultant vessel injury and distal embolization of the disrupted clot make these devices less favorable in the setting of acute IS.

## Thrombus Aspiration

Mechanical aspiration of a clot from the intracranial arteries appears as an attractive and safe therapeutic option. The Penumbra embolectomy system consisted of an aspiration catheter and a separator wire, which was advanced over a guidewire to the site of arterial occlusion. The Penumbra system was been investigated in several single-center and multicenter trials. The Penumbra pivotal stroke trial [56] prospectively evaluated 125 acute IS patients from 24 US and Europe centers, presenting within 8 hours of symptom-onset. This study used the MERCI trial data for historical control with the aim of proving equivalence. Recanalization was achieved in 81.6% of patient. However, despite the relatively high recanalization rate, favorable clinical outcome was achieved in only 25% of all patients. Furthermore, even after successful recanalization, only 29% patients achieved favorable outcome at 3 months. Overall mortality was 32.8% and SICH occurred in 11.2%. However, some recent single-center studies reported better favorable outcomes results with the Penumbra system as successful recanalization in 93%, good clinical outcome in 48% and only 11% mortality [57]. The advantage of the Penumbra device is that it operates from the proximal end, minimizing the need to blindly penetrate into the occluded vascular segment of the clot. It can achieve arterial recanalization without the use of adjunctive thrombolytics, facilitating single access platforms. Due to the flexibility and variety of available sizes (0.26 - 0.54 inch) of the reperfusion microcatheters, even smaller distal arterial branches can be successfully accessed.

## Thrombus Entrapment

Self-expandable stents (SES) were initially approved for vessel remodeling in the treatment of cerebral aneurysms and intracranial atherosclerotic disease. In acute IS, these devices use a stent to recanalize the occluded artery and trap the clot. Furthermore, stents could be used to remove the clot in a proximal artery (cervical ICA) and then navigate the device distally to trap the clot from intracranial tandem occlusion. Self-expandable stents are becoming increasingly popular because of their flexibility and ease of navigation, especially in the complex intracranial circulation. They include the Neuroform stent (Boston Scientific, Natick, MA), Enterprise stent (Cordis, Miami Lakes, FL), Solitaire/Solo stent (ev3, Irvine, CA) and the Wingspan stent (Boston Scientific). The first three stents were initially approved for stent-assisted coiling of wide-neck aneurysms while Wingspan was approved for atherosclerotic intracranial disease.

Intracranial stent use is associated with the risk of stent-thrombosis, requiring long-term platelet inhibition. In the acute setting, administration of GPIIb/IIIa inhibitors or antiplatelet agents may increase the risk of hemorrhagic transformation. Similarly, patients treated with IV and/or IA thrombolytics have a much higher risk of bleeding [58]. Moreover, stent placement in acute IS may be considered an un-necessary permanent implant to resolve a temporary occlusion, inviting long-term problems associated with it [59]. Temporary and retrievable stents are a good alternative to temporarily scaffold the lesion for flow restoration and removal of the thrombus.

Phatouros et al reported the first endovascular stenting of an acutely occluded basilar artery that could not be recanalized with IA thrombolysis and balloon angioplasty. The deployment of a balloon-mounted stent resulted in complete recanalization [60]. Currently used stents in acute IS are primarily coronary balloon-mounted self expandable stents such as the Neuroform (Boston Scientific, Natick, Massachusetts), Wingspan (Boston Scientific), Enterprise (Codman Neurovascular, Raynham, Massachusetts), Solitaire (ev3, Irvine, California), or the Leo SES (Balt, Montmorency, France). Neuroform and Wingspan stents have an open-cell design whereas Enterprise and Leo stents are closed-cell design. The closed- cell design allows resheathing of the stent after partial deployment [61]. Stent-Assisted Recanalization in Acute Ischemic Stroke (SARIS) trial was the first FDA approved prospective pilot study of direct self-expanding stent placement in acute IS patients who failed IV-tPA or had a contraindication to it [62]. The study included 20 patients presenting within 6 hours of symptom-onset and achieved 100% recanalization rate with adjuvant therapies such as angioplasty, IV-tPA and IA-tPA. Reduction in NIHSS score by at least 4 points was seen in 65% patients. The study reported 1-month mortality in 25% patients while moderate clinical outcome was noted in 60% patients.

Enterprise, Neuroform, and Wingspan SES systems have been found to have better navigability, cause less vasospasm and side-branch occlusions than balloon-mounted stents. Additionally, SESs do not require balloon inflation in vessels with an unknown diameter. However, SES can only be used in the arteries with diameter larger than 2 mm and chemical thrombolysis is often required to achieve complete distal recanalization. Fitzsimmons and Nelson reported the deployment of Neuroform stent in an occluded left middle cerebral artery after IA infusion of a glycoprotein (GP) IIb/IIIa inhibitor failed to recanalize the vessel [63]. SESs are preferred over balloon-mounted stents and the reported recanalization rates vary between 79% and 92%, with moderate clinical outcome in up to 50% of patients [64]. In acute IS, Neuroform and Wingspan stents achieved recanalization in 67-89% patients, with very low rates of in-stent restenosis during 9 months of follow up [65].

Retrievable stents have a cylindrical form and contain cell struts. They are placed over the clot which promotes immediate reperfusion. They are deployed either as temporary bypass to optimize the surface area of IA thrombolysis or direct mechanical thrombectomy. There are no studies comparing these two approaches. In the temporary bypass technique, the stent facilitates IA thrombolysis as the fibrinolytic drugs can be administered through a guide catheter or a second microcatheter and the stent is removed after recanalization is achieved. It consists of deploying the stent over the clot and waiting for 5 minutes for embedding the clot in the stent before retrieval. Body temperature causes the nitinol stent to increases its radial force and facilitates clot retrieval. After deployment, the stent is then recovered into the guiding catheter along with the clot in tow.

The Solitaire device was the first retrievable stent used for acute IS. It was initially designed as a detachable stent and approved for aneurysm bridging treatment. Same concept underwent modifications in the stent cell design, material and distal tip. MindFrame company developed the Capture systems while Concentric launched Trevo device in 2010. The Trevo system is a non-deployable stent-like device with a soft malleable body to allow easy navigation through tortuous vessels while its distal closed-end prevents vessel perforation. The device can be used even in small arteries measuring 1.5 to 3.5 mm in diameter. A single-center prospective pilot study on 20 patients with an acute IS in the anterior circulation, presenting within 8 hours from symptom-onset, showed successful revascularization in 90% arteries with the Solitare device, mRS score of ≤ 2 was achieved by 45% subjects at 3 months [66]. The rescue, combined and stand-alone thrombectomy (RECOST) study was performed to evaluate and appraise the timing, safety and efficacy of an integrated stroke management protocol with the Solitaire device. Complete recanalization could be achieved in 84% patients, with 2% SICH rates and 54% of patients achieved mRS ≤ 2 at 3 months [67]. The use of retrievable stents does not require an overly large interventional experience. The procedural time taken to recanalize the occluded intracranial artery ranges from 37 to 88 minutes, relatively lower than the multi-Merci (96 minutes) and Penumbra Pivotal (97 minutes) devices [68]. The mortality rate is achieved with retrievable stents is also relatively lower and ranges between 0% and 27%.

The recently conducted SOLITAIRE FR with the intention to treat for thrombectomy (SWIFT) trial was a multicenter, randomized controlled study of 144 acute IS patients. Subjects were randomized to either MERCI or SOLITAIRE treatment. Recanalization was demonstrated in 60.7% of patients with SOLITAIRE device as compared to only 24.1% with the MERCI. Patients treated with the SOLITAIRE device had lesser SICH, reduced mortality and better outcomes at 3 months than MERCI [69].

Many clinical trials involving retrievable stents are currently running. The Trial and Cost Effectiveness Evaluation of IA Thrombectomy in acute IS (THRACE) study will compare IV thrombolysis with thrombectomy procedures (Merci retriever, Penumbra system, Catch device, and the Solitaire FB stent) in 480 patients. The Thrombectomy Revascularization of Large Vessel Occlusions (TREVO 2) study in the United States aims at enrolling 178 patients to compare the Trevo system with Merci retriever.

## Multimodality Treatment

Newer acute stroke units, have the ability to treat acute stroke patients with different complementary modalities such as injecting a bolus dose of IV-tPA while awaiting the endovascular suite preparation. Although a theoretical possibility of increased bleeding and puncture site hematoma exists, the data from coronary artery revascularization trials appears.

In the interventional management of stroke (IMS) study, patients with NIHSS score of > 10 were treated initially with 0.6 mg of IV-tPA followed by IA-tPA (upto 22 mg) [70]. Partial or complete re-canalization was achieved in 64% of patients after IA treatment. There was no control arm in the study but compared with historical controls treated with IV-tPA, the IV and IA tPA treated patients showed only a modest trend to improved clinical outcomes. The IMS II study was an open-labeled single-arm pilot study that evaluated combined IV/IA therapy and low-energy sonography via the EKOS Primo sonography microcatheter in patients with acute large IS (NIHSS ≥ 10) treated within 3 hours of symptom-onset [71, 72]. Complete re-canalization occurred in 41.4% of sonography microcatheter-treated occlusions at 60 minutes. Complete re-canalization rates at 2 hours or at the end of the procedure were 68.9% in the ultrasound catheter treated group. EKOS Primo sonography microcatheter demonstrated a trend toward improved recanalization rates compared with a standard microcatheter. The IMS III trial was designed to study clinical outcomes with a reduced dose of IV-tPA before attempting IA recanalization. The trial was stopped prematurely stopped after an interim analysis that showed the futility of continuing further since it would not have met its predefined superiority outcomes. Importantly, no significant increase in the adverse outcomes due to the combined approaches was observed. The ongoing Thrombectomy in Unsuccessful Stroke Thrombolysis (THRUST) trial is comparing the effect of thrombectomy with the Merci Retriever versus no intervention following unsuccessful IV thrombolysis. The THRUST study is part of the international collaboration Safe Implementation of thrombolysis in stroke (SITS) [73].

The RECANALISE study showed that a combined IV-endovascular approach was associated with higher recanalization rates than IV-tPA alone. The study showed significantly higher rates of recanalization with the combined approach (87%) as compared to 52% with IV-tPA alone (RR 1.49; 95% CI, 1.21 - 1.84; P < 0.0002). Recanalization was associated with better neurologic improvement at 24 hours in the dual approach group versus 39% in the IV group. Mortality rates (17%) at 90 days did not differ between the two groups. SICH rates in the combined IV-endovascular and the IV alone group were 9% and 11%, respectively [74]. In a recent retrospective analysis of 1,122 patients with anterior circulation IS treated at 13 centers with IA thrombolysis and mechanical thrombolysis within 8 hours of symptom-onset, it was found that the multimodal therapy was associated with higher recanalization rates (74%) compared with pharmacologic therapy (61%) or mechanical therapy (63%, P < 0.001) [75]. The multimodal therapeutic approach was found to be superior even in patients with the intracranial ICA acute occlusions, often associated with poor prognosis.

## Discrepancies and Alternatives

IA thrombolysis and mechanical strategies are able to achieve high rates of arterial recanalization. However, a successful recanalization in acute IS does not always translate into good neurological recovery. In fact, the rates of recanalization are almost 3 times higher than the absolute increase in good (14.8%) or excellent (13.0%) clinical outcomes [76]. However, only a few studies have formally monitored arterial recanalization. Furthermore, the diagnostic modalities to assess recanalization have been variable (Table 2). The rate of recanlaization and its association with functional outcome and SICH in some of the studies are shown in Table 3. Owing to the heterogeneity of patient population and the time of intervention, this comparison may not be considered as proper by some critiques. The disparity in clinical outcomes is due to either limitations in the outcome measurement tools to adequately detect real clinical improvement in stroke victims or completion of stroke injury before revascularization and reperfusion. This discrepancy increases with the longer time window. Perhaps, image-guided therapy could help in better selection of the patients and improve these results. Another likely explanation lies in the inter-individual variability in the presence of good collateral cerebral circulation beyond an occlusion during the first few hours after acute IS. An efficient collateral circulation may potentially sustain tissue viability until recanalization occurs and such patients might benefit even when treated beyond the currently recommended therapeutic window. However, the current methods of evaluating collaterals in acute IS are not well developed.

**Table 2:** Arterial Occlusions and Recanalization Patterns in Some Clinical Trials in Acute Ischemic Stroke

TRIAL	Treatment	Number of patients	Highest reported recanalization rate (%)	Assessment of recanalization
NINDS [9] Active	IV-tPA	312	--	
Control	Placebo	312	--	None
ECASS-3 [10] Active	IV-tPA	418	--	None
(3 - 4.5 hours) Control	Placebo	403	--	
DIAS (3 - 9 hours) [18] Active	IV-Desmotepase	71 (Total)	49	
Part-1 25 mg		16	56	
37.5/50 mg		13	46	
Part-2 62.5 µg/kg		13	23	MRA-complete or partial recanalization
90 µg/kg		15	47	
125 µg/kg		14	71	
Control		26	19	
Part-1	Placebo	16	19	
Part-2		10	20	
DIAS-II (3 - 9 hours) [20] Active	IV-Desmoteplase			
90 µg/kg		57		
125 µg/kg		66		
Control	Placebo	63		
Active PROACT-II [35]	IA r-proUK	121	66	Cerebral Angiography (TIMI 2 + 3)
(TIMI 2 + 3) Control	Placebo	59	18	
Active	IV + IA TPA	11	81	
				Cerebral Angiography
IMS [69] Control	Placebo + IA TPA	10	50	
Active	MERCI ± Lytic			
MCA		80	45	Cerebral Angiography (any recanalization)
ICA		47	53	
MERCI [44] VA/BA		14	50	
Control	--	59	18	
	Placebo cases from PROACT-2			
Active	IV-tPA + TCD	63	38	
CLOTBUST [28] (at 2 hours) Control	IV-tPA	63	13	TCD
Active	IV-tPA + Ultrasound	14	29.3	
TRUMBI [29] Control	IV-TPA	12	50.0	MRA

**Table 3:** Comparative Rates of Recanalization, Symptomatic Intracranial Hemorrhage and Functional Outcome in Various Clinical Trials

Revascularization approach	Partial and incomplete recanalization (%)	SICH	mRS 0 - 2 at 3 months
IV tPA < 3 hrs CLOTBUST [28], TUCSON [80]	40	4.4	39
IA Pro-urokinase < 6 hrs PROACT II trial [35]	66	10	40
IV tPA < 3 hrs + IA tPA IMS II trial [70]	64	9.9	38
Merci^TM^ < 8 hrs [44], Multi-Merci Study [50]	57	9.8	36
Penumbra^TM^ < 8 hrs Penumbra clinical study [55]	82	11.2	25
IV tPA sonothrombolysis < 3hrs CLOTBUST tPA + TCD arm [28]	83	3.8	51

tPA: tissue plasminogen activator; SICH: symptomatic intracranial hemorrhage; mRS: modified Rankin Scale; PRO-UK: Pro-Urokinase.

## Alternative Reperfusion Strategies

Although not strictly thrombolysis, cerebral reperfusion during acute IS can be augmented via some alternative strategies using an anterograde or retrograde route. Anterograde reperfusion can be expedited by raising the systemic blood pressure through the use of vasopressors leading to global reperfusion. Retrograde reperfusion can be facilitated with a transarterial or transvenous approach. The transarterial approach involves the endovascular deployment of the NeuroFlo device (CoAxia, Maple Grove, MN). This dual balloon catheter partially occludes the aorta above and below the level of the renal arteries to divert blood flow from the systemic circulation toward the cerebral circulation [77]. The Safety and Efficacy of NeuroFlo Technology in Ischemic Stroke (SENTIS) trial was completed in 2010. This study enrolled 515 patients from 68 centers in 9 countries. Although, there was no statistical significance in disability at 90 days, (OR, 1.17; CI, 0.81 - 1.67; P = 0.407), the device did not cause any increase in the serious adverse events (P = 0.923). A non-significant trend for improvement on all-cause mortality, in patients presenting early, older than 70 years or with moderate strokes was observed [78]. Transvenous retrograde reperfusion is an experimental technique that diverts blood from the femoral artery into the transverse venous sinuses via transvenous catheters, in an attempt to reduce the ischemic penumbra and improve neurologic outcomes [79]. Recently, Ribo et al described a novel and imaginative method in which oxygenated blood was perfused through the occluding clot [80]. A microcatheter was passed through the clot and 20 mL of oxygenated blood was injected over 2 minutes beyond occlusion, to “buy time” until definitive reperfusion could be achieved. Further investigational human trials are required before introducing such a novel concept to current stroke therapies.

## Future Directions

Acute ischemic stroke continues to be a devastating disease. Early access to definitive therapy remains the key for a better outcome. The search for newer, more effective and yet, safer pharmacological, interventional, and combined treatments continues. Acute invasive interventional strategies continue to improve along with the noninvasive (medical or mechanical adjunctive) modalities. Both approaches appear promising. While the former approach is expected to become the primary therapeutic approach at tertiary care and fully equipped centers, the latter approach would remain the mainstay of acute stroke treatment at primary stroke centers and centers with limited access to catheter insertion. Therefore, the reperfusion therapy would begins with noninvasive strategies in most of the acute IS patients and, carefully selected patients may later be subjected to various endovascular treatments, maximizing the chances of recanalization. The diversity of new emerging approaches makes stroke an exciting and rapidly evolving field.
